# Author Correction: Spatial transcriptomics uncover sucrose post-phloem transport during maize kernel development

**DOI:** 10.1038/s41467-023-44536-w

**Published:** 2024-01-04

**Authors:** Yuxin Fu, Wenxin Xiao, Lang Tian, Liangxing Guo, Guangjin Ma, Chen Ji, Yongcai Huang, Haihai Wang, Xingguo Wu, Tao Yang, Jiechen Wang, Jirui Wang, Yongrui Wu, Wenqin Wang

**Affiliations:** 1 College of Life Science, Shanghai Normal University, 100 Guilin Road, Shanghai, 200233 China; 2 National Key Laboratory of Plant Molecular Genetics, CAS Center for Excellence in Molecular Plant Sciences, Shanghai Institute of Plant Physiology & Ecology, Shanghai, 200032 China; 3 Triticeae Research Institute, Sichuan Agricultural University, Chengdu, 611130 China; 4 University of the Chinese Academy of Sciences, Beijing, 100049 China; 5 State key Laboratory of Crop Gene Exploration and Utilization in Southwest China, Sichuan Agricultural University, Chengdu, 611130 China

**Keywords:** Seed development, Plant molecular biology, Transcriptomics

Correction to: *Nature Communications* 10.1038/s41467-023-43006-7, published online 8 November 2023

The original version of this Article contained an error in Figure 1a. The panels representing kernel sections for S18D_1 and S24D_1 were mistakenly swapped. This has been corrected in both the PDF and HTML versions of the Article.

