# Supplementary material for: Spatial transcriptomics uncover sucrose post-phloem transport during maize kernel development
Source: Nat Commun. 2023 Nov 8;14:7191. doi: 10.1038/s41467-023-43006-7 (PMC10632454; doi:10.1038/s41467-023-43006-7)
Supplement: Supplementary file 1 — Supplementary Information [file 41467_2023_43006_MOESM1_ESM.pdf]

SUPPLEMENTARY INFORMATION

Spatial Transcriptomics Uncover Sucrose Post-Phloem Transport During Maize Kernel Development

Yuxin Fu[1,2,3#], Wenxin Xiao[1#], Lang Tian[1], Liangxing Guo[2], Guangjin Ma[2,4], Chen Ji[2,4], Yongcai Huang[2,5], Haihai Wang[2], Xingguo Wu[1], Tao Yang[2,5], Jiechen Wang[2], Jirui Wang[3,5], Yongrui Wu[2*], Wenqin Wang[1*]

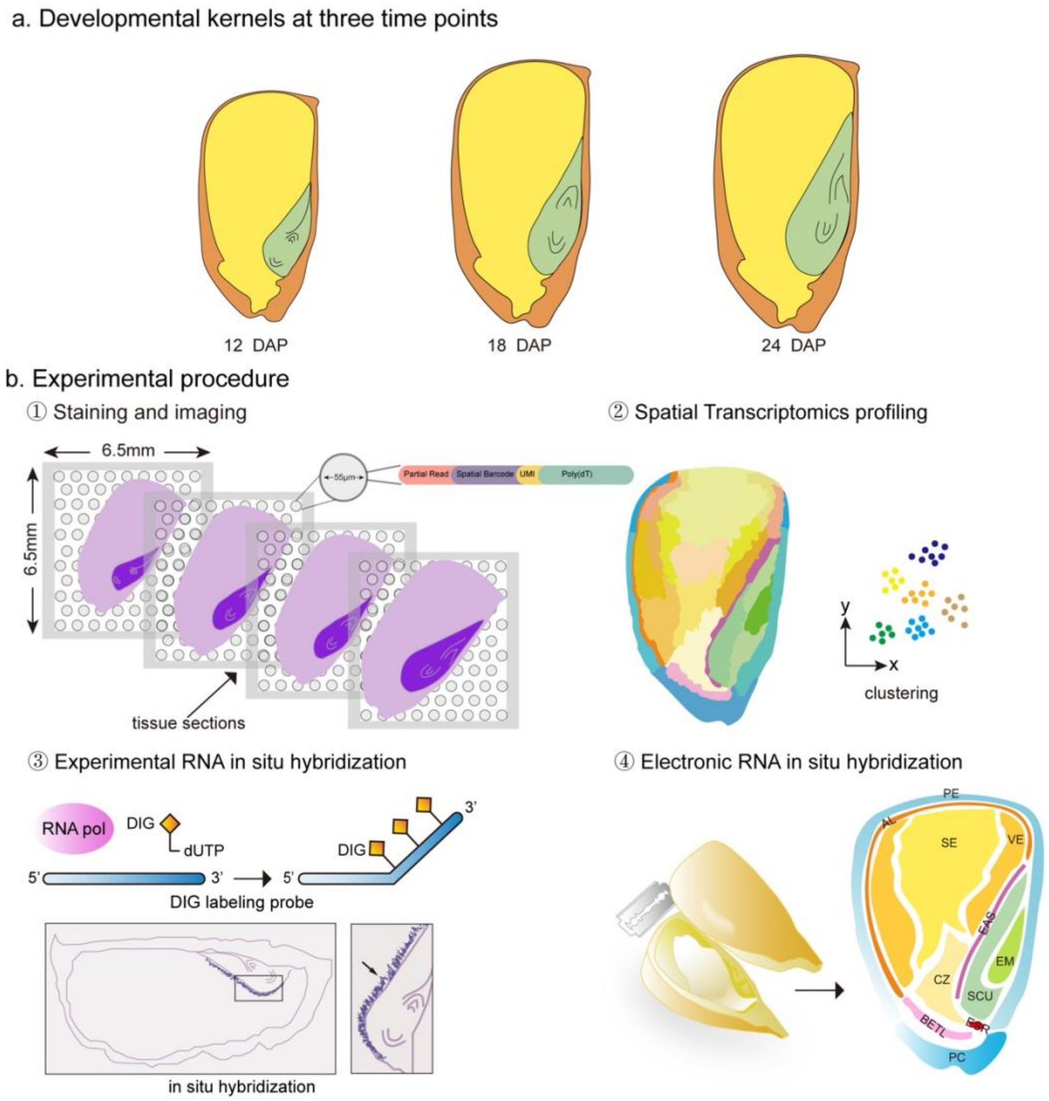

Figure S1. Overview of the spatial transcriptomic study.

a. Developmental kernels at three time points of filling stages are included. Time points are defined as 12, 18 and 24 days after pollination (DAP).

b. Experimental procedure: ① The sample is subjected to embed, section, fix, hematoxylin & eosin stain and image. The capture area of Visium chip has a side length of 6.5 mm. Each square area includes 5,000 barcoded spots with a diameter of 55 μm. Each spot contains millions of oligonucleotides that are unique to that spot; ② Spatial transcriptomics profiling includes library construction, sequencing and bioinformatically clustering; ③ Verification using RNA in situ hybridization; ④ Generating spatial gene expression atlas.

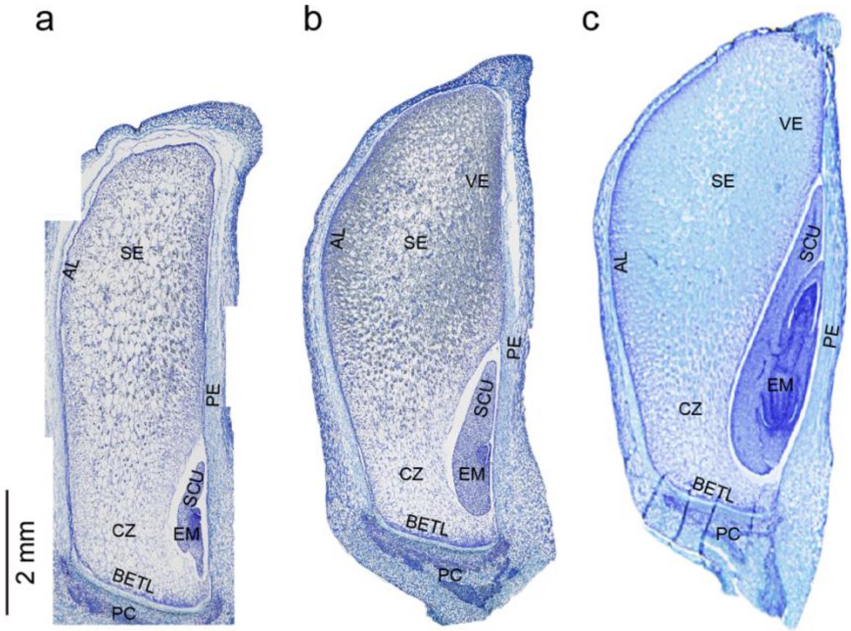

Figure S2. Light microscopy of semi-thin kernels at 10, 14 and 18 DAP.
a, 10 DAP; b, 14 DAP; c, 18 DAP.
The histological images show the maternal-derived regions: placento-chalazal (PC) and pericarp (PE); the endosperm compartments: basal endosperm transfer layer (BETL), conducting zone (CZ), starchy endosperm (SE), vitreous endosperm (VE), aleurone (AL); and the embryo regions: scutellum (SCU) and embryo meristem (EM). Scale bar is 2 mm.

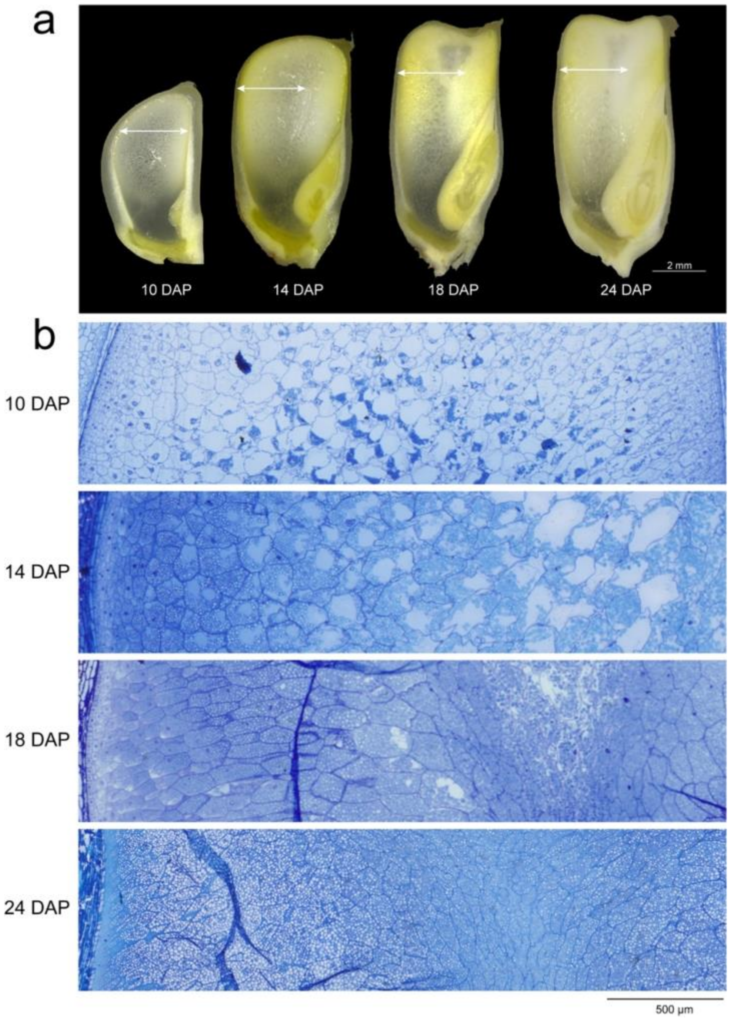

Figure S3. Storage metabolite accumulation and cell size measurement during maize endosperm development

a, Longitudinal free-hand sections of developing W64A kernels. The double-headed arrow line indicates the regions across the endosperm where semi-thin section is checked. Scale bar is 2 mm.

b, Light microscopy of semi-thin sections for the developing W64A endosperms at 10, 14, 18, 24 DAP. Scale bar is 500 µm.

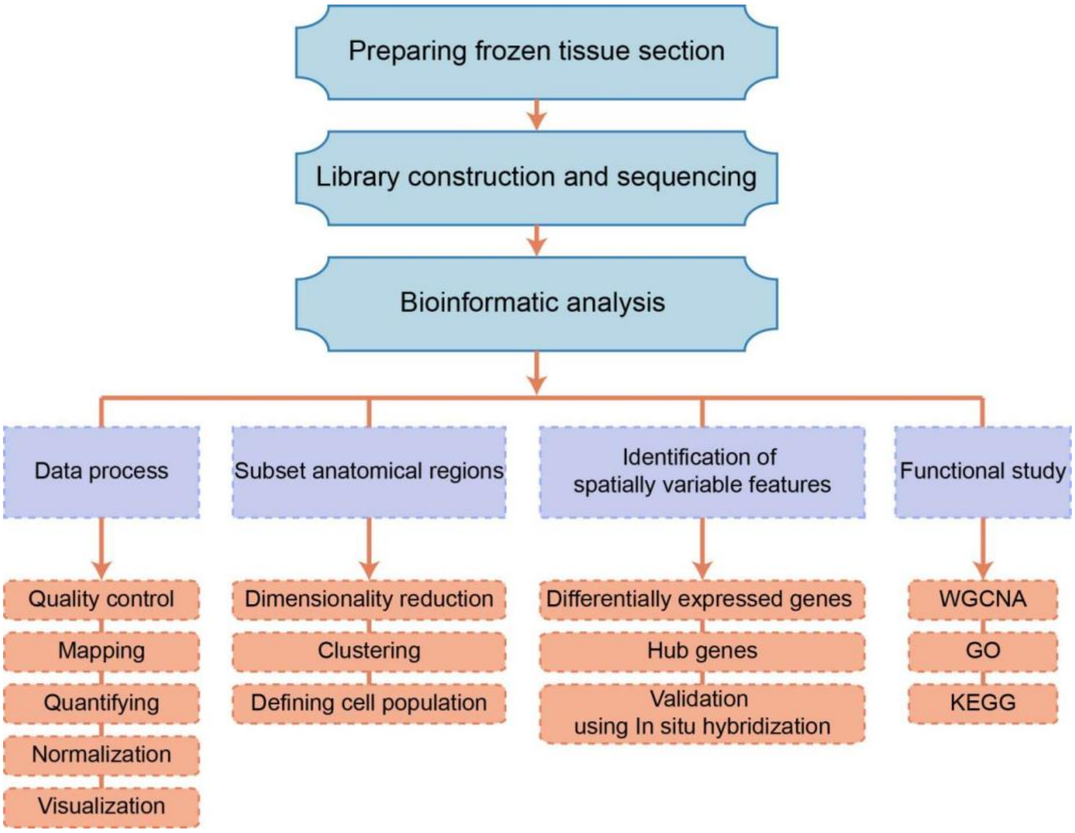

Figures S4. The analysis pipeline for spatial transcriptomic study.

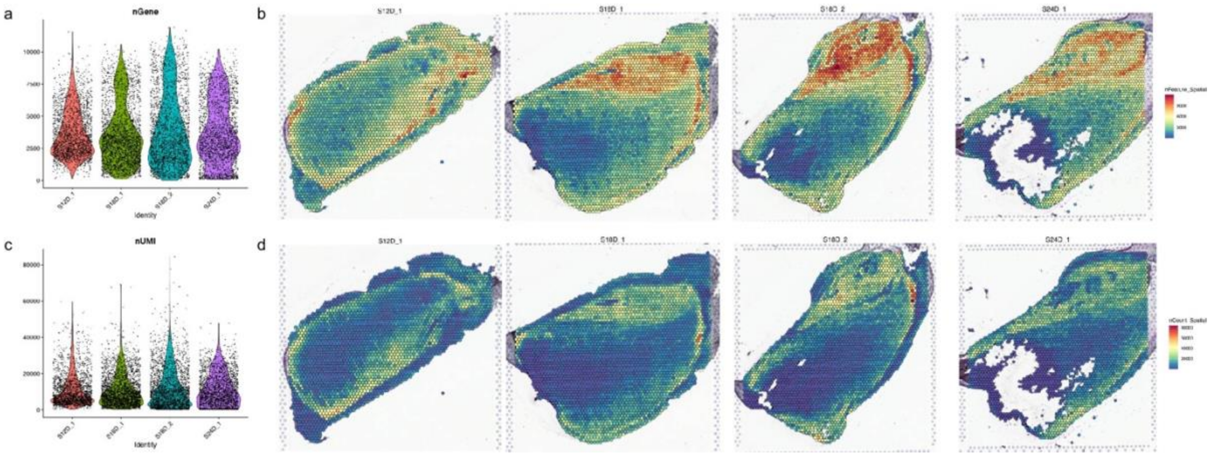

Figure S5. The density of expressed genes and transcripts in a spot.
a. The number of expressed genes in a spot.
The y-axis represents the number of genes expressed in a spot.
b. The spatial distribution of expressed genes on the sample sections.
c. The numbers of RNA molecules with Unique Molecular Identifiers (UMI) in a spot.
The y-axis represents the number of transcripts in a spot.
d. The spatial distribution of the unique transcripts in the sample sections.
The color changes gradually from green, yellow to red when the density gradually increases.

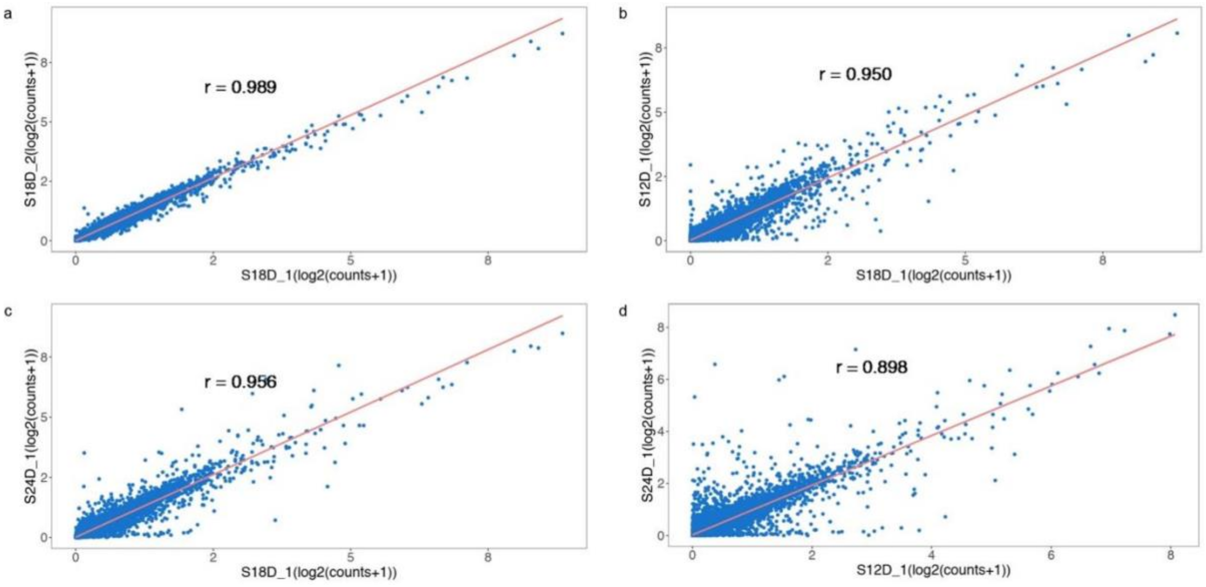

Figure S6. Pearson correlation among samples.
Scatterplot showing Pearson correlation between aggregated gene counts from samples. The biological replicates (S18D_1 and S18D_2) show the best correlation compared to what was observed for the other samples.
a. Scatterplot of S18D_1 and S18D_2.
b. Scatterplot of S18D_1 and S12D_1.
c. Scatterplot of S18D_1 and S24D_1.
d. Scatterplot of S12D_1 and S24D_1.

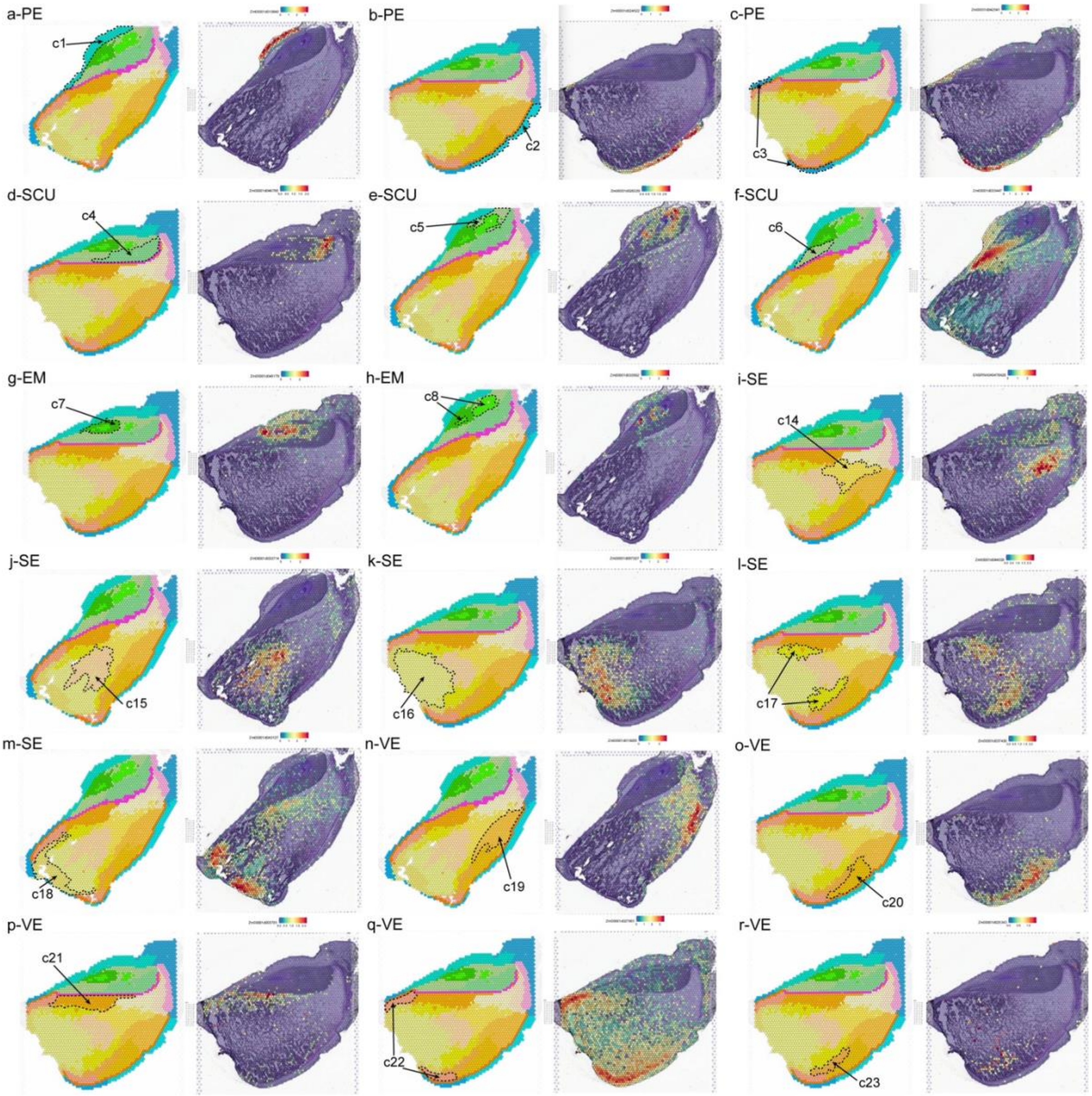

Figure S7. Representatives of marker genes defined by spatial transcriptomic data in the tissues of pericarp, embryo and endosperm.

The left panel (e.g., a-PE) provides an overview of the 25 cell populations, while the right panel (e.g., a-PE) displays a snapshot of the spatial expression of marker gene. The abbreviations PE, SCU, EM, SE, and VE correspond to pericarp, scutellum, embryo meristem, starchy endosperm, and vitreous endosperm, respectively.

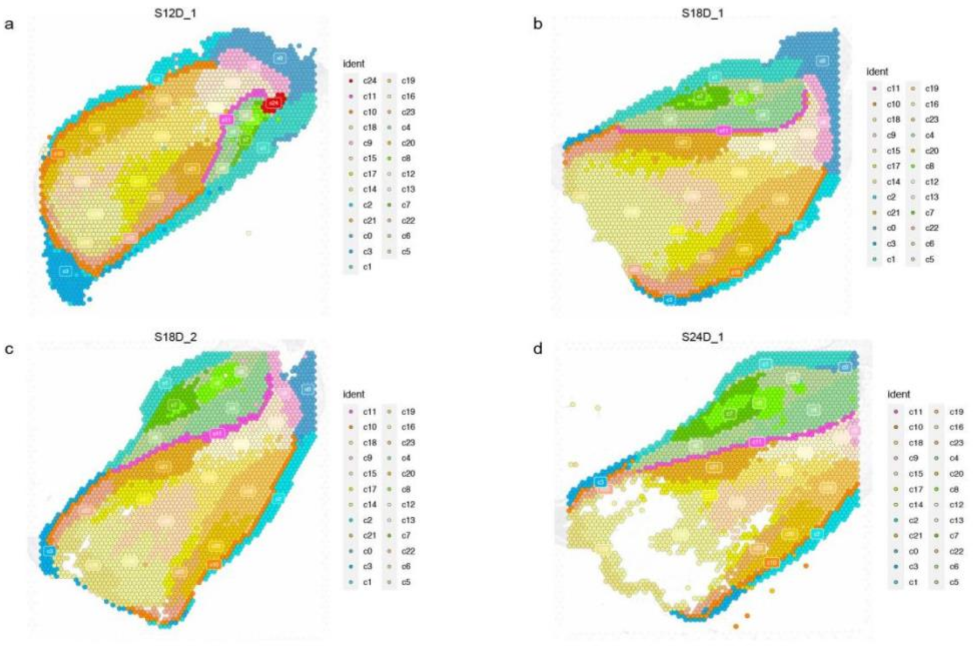

Figure S8. The defined clusters after dimensional reduction.
The 25 clusters from dimensional reduction are mapped back to tissue sections, showing their location on the kernel. Each dot in the figure represents a tissue spot containing one to three cells. The clusters are distinguished by different colors.

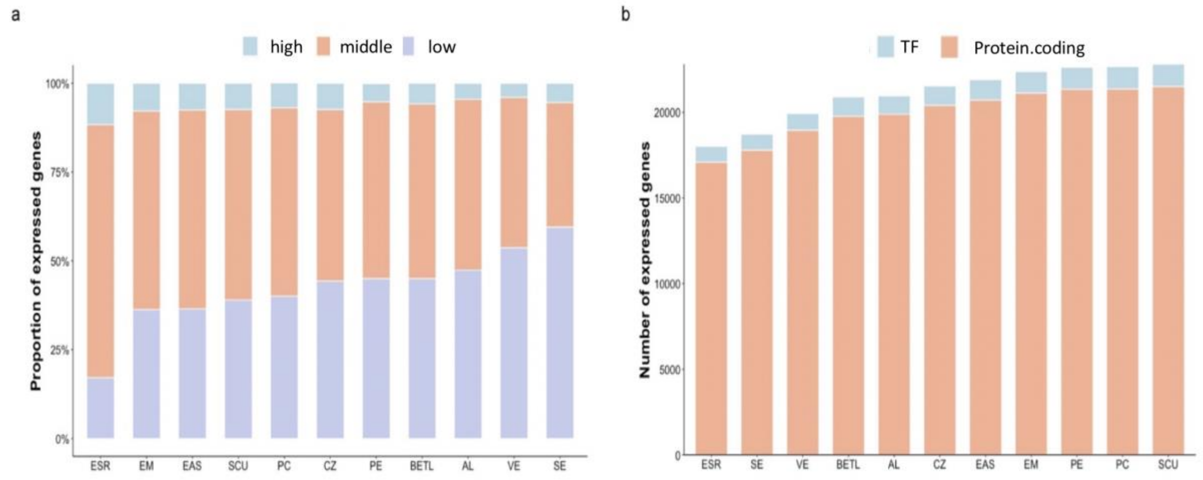

Figure S9. Summary of gene expression in kernel compartments.
a. The gene numbers expressed at different level (based on UMI index) in kernel compartments.
b. The gene numbers of protein coding genes and transcription factors (TF) in kernel compartments.

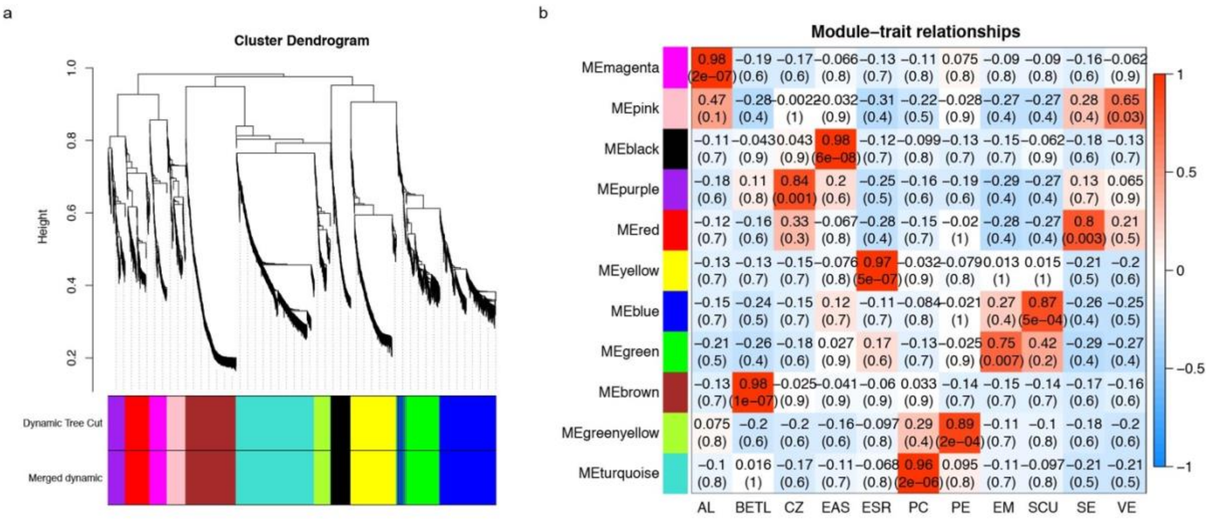

Figure S10. The gene co-expression networks using WGCNA in maize kernel.
a. Gene clustering dendrogram.
b. The correlations between detected modules and kernel compartments hierarchically clustered based on Euclidean distance. The progressively saturated red colors indicated higher overlap among the functional modules.

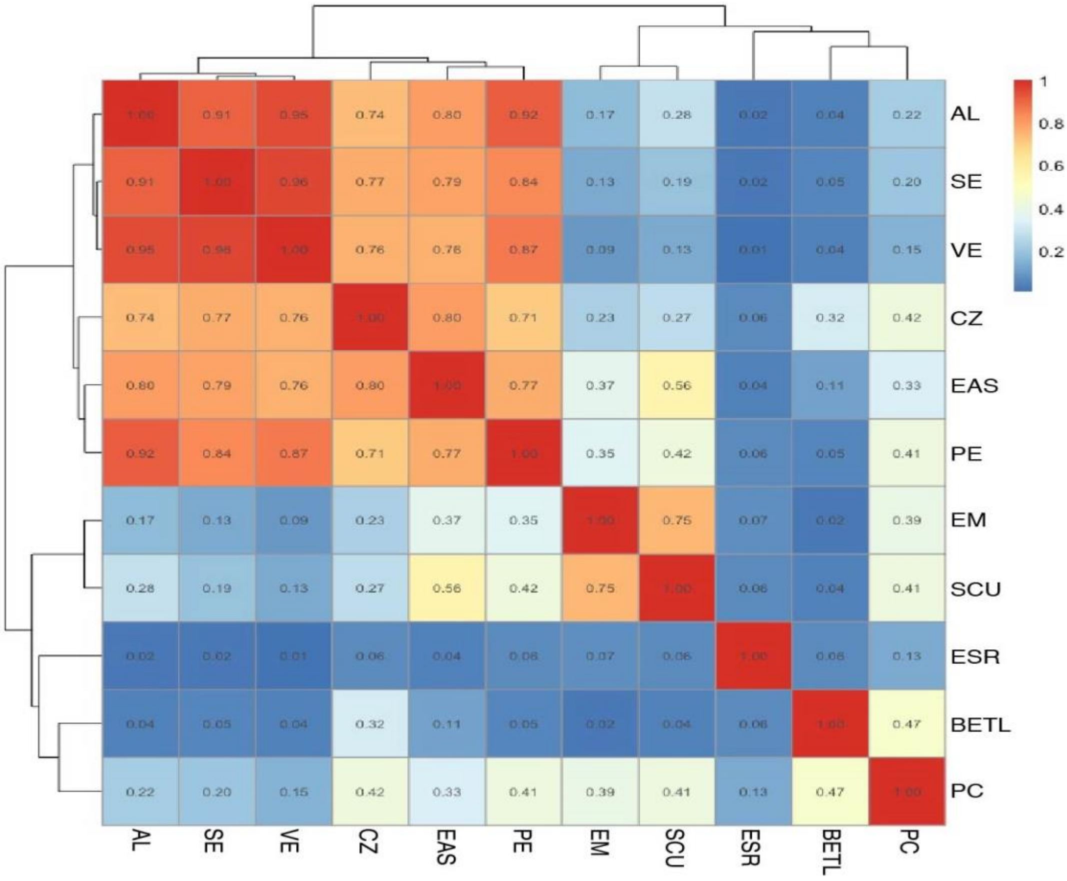

Figure S11. Pairwise Pearson correlation analysis over kernel compartments.

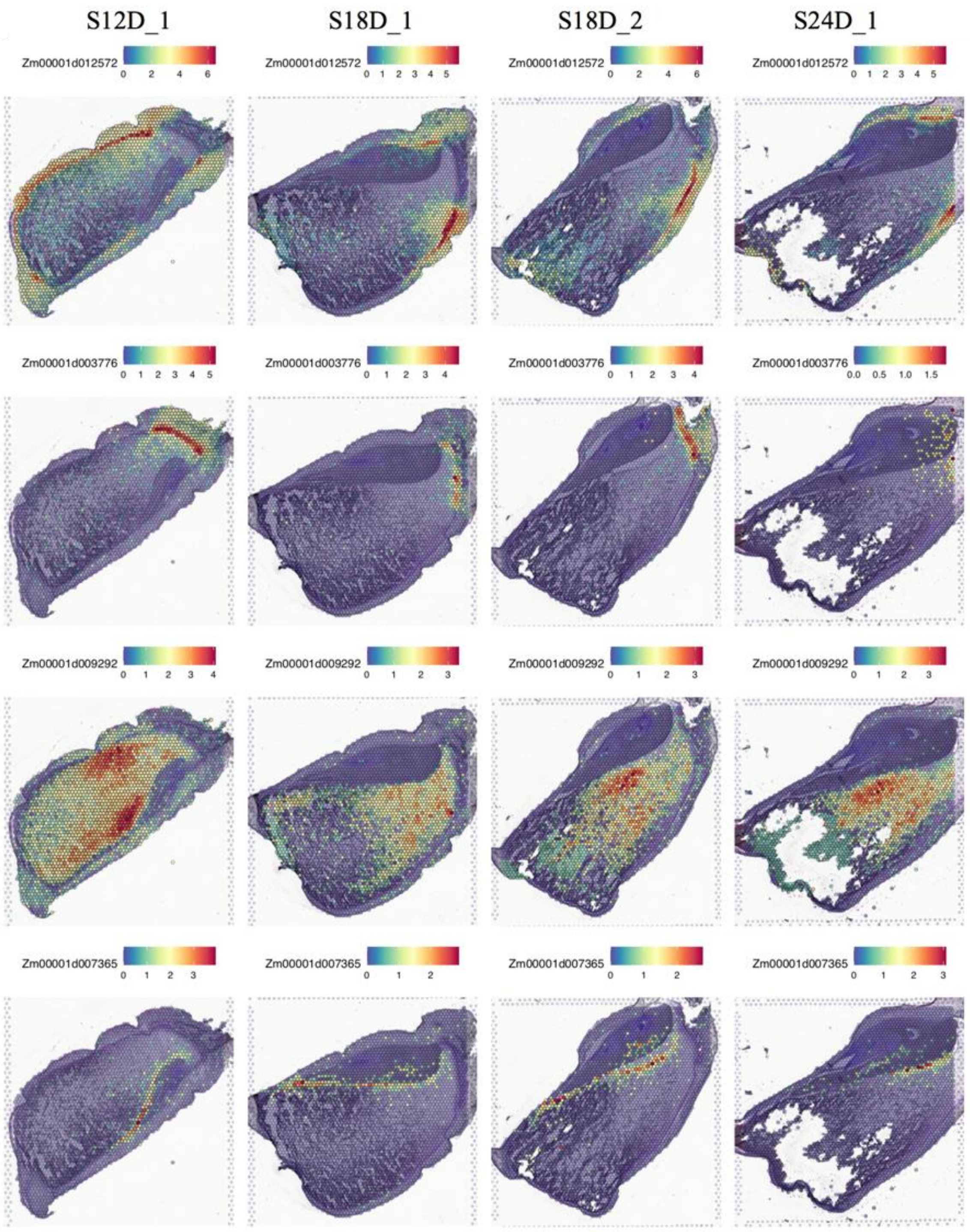
S12D_1
S18D_1
S18D_2
S24D_1
Zm00001d012572
Zm00001d003776
Zm00001d009292
Zm00001d007365

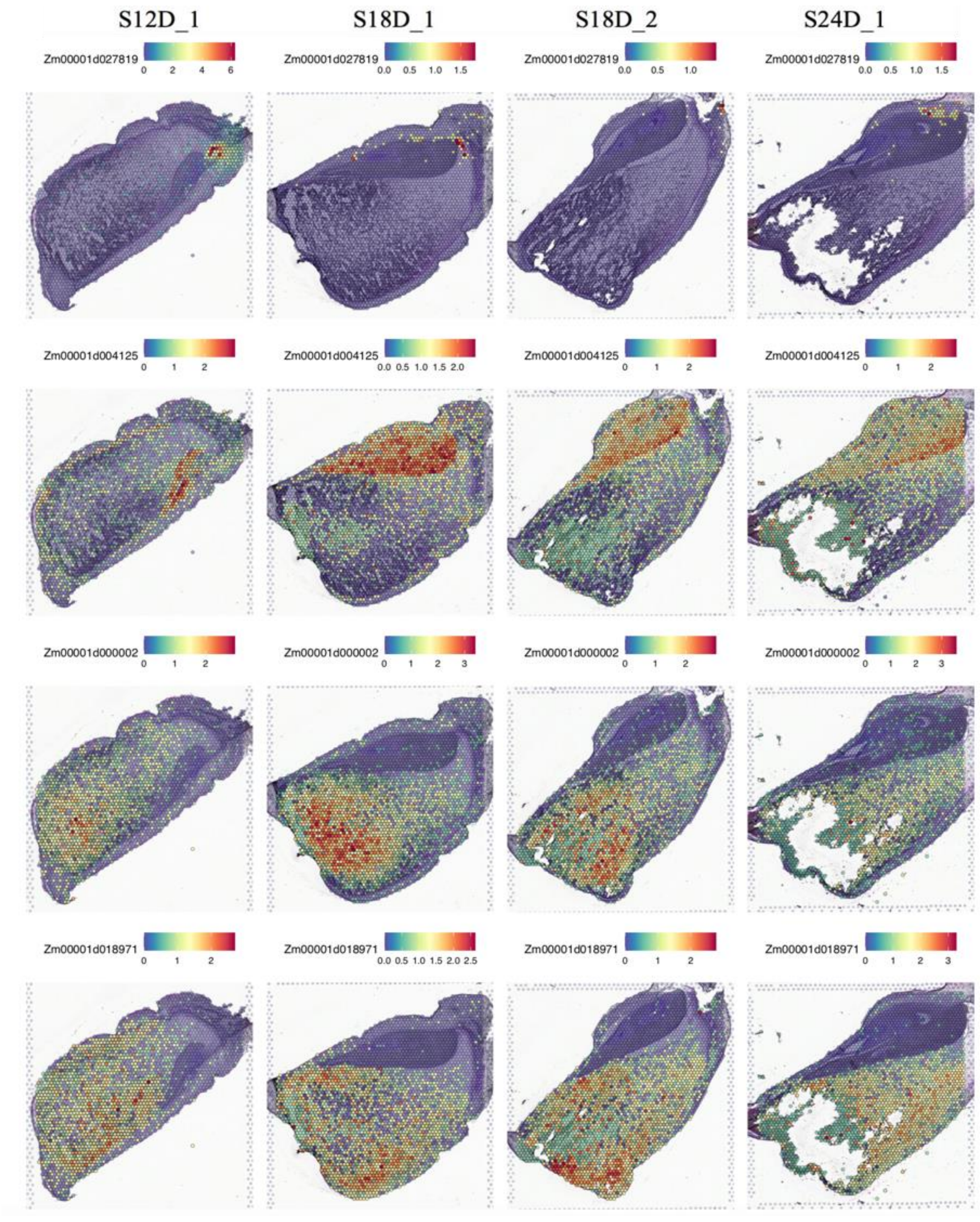

Figure S12. Representatives of electronical RNA in situ hybridization using known markers that are consistent with the previous studies (Li, 2014; Zhan, 2015).

We selected one marker from each compartment as following except EM, PC and PE due to unavailability from other research: AL9 in aleurone (Zm00001d012572); CWIN2 in BETL (Zm00001d003776); FL3 in CZ (Zm00001d009292); SWEET14a in EAS (Zm00001d007365); ESR2 in ESR (Zm00001d027819); ACCase in SCU (Zm00001d004125); SS2 in SE (Zm00001d000002); O2 in VE (Zm00001d018971).

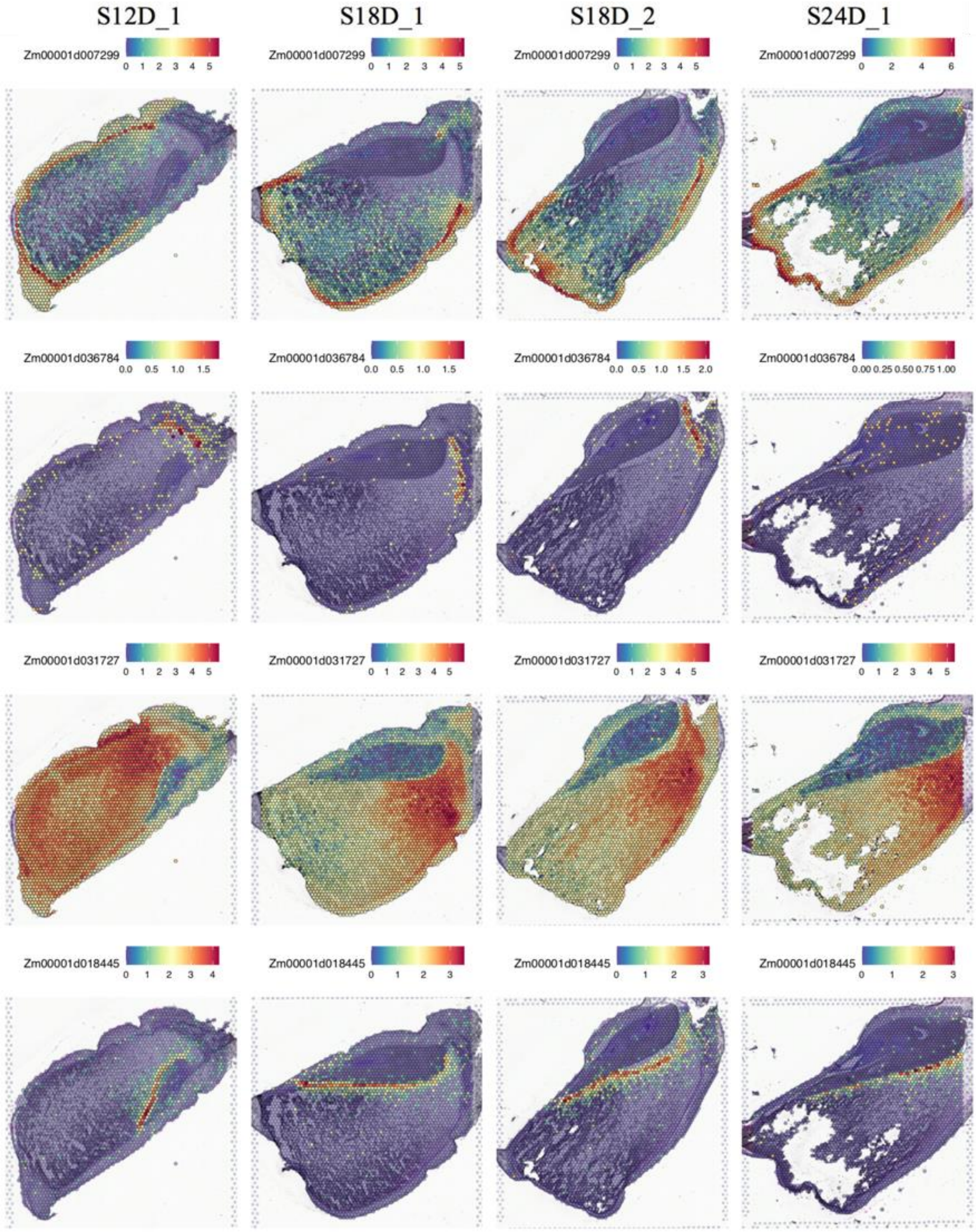
S12D_1
S18D_1
S18D_2
S24D_1
Zm00001d007299
Zm00001d036784
Zm00001d031727
Zm00001d018445

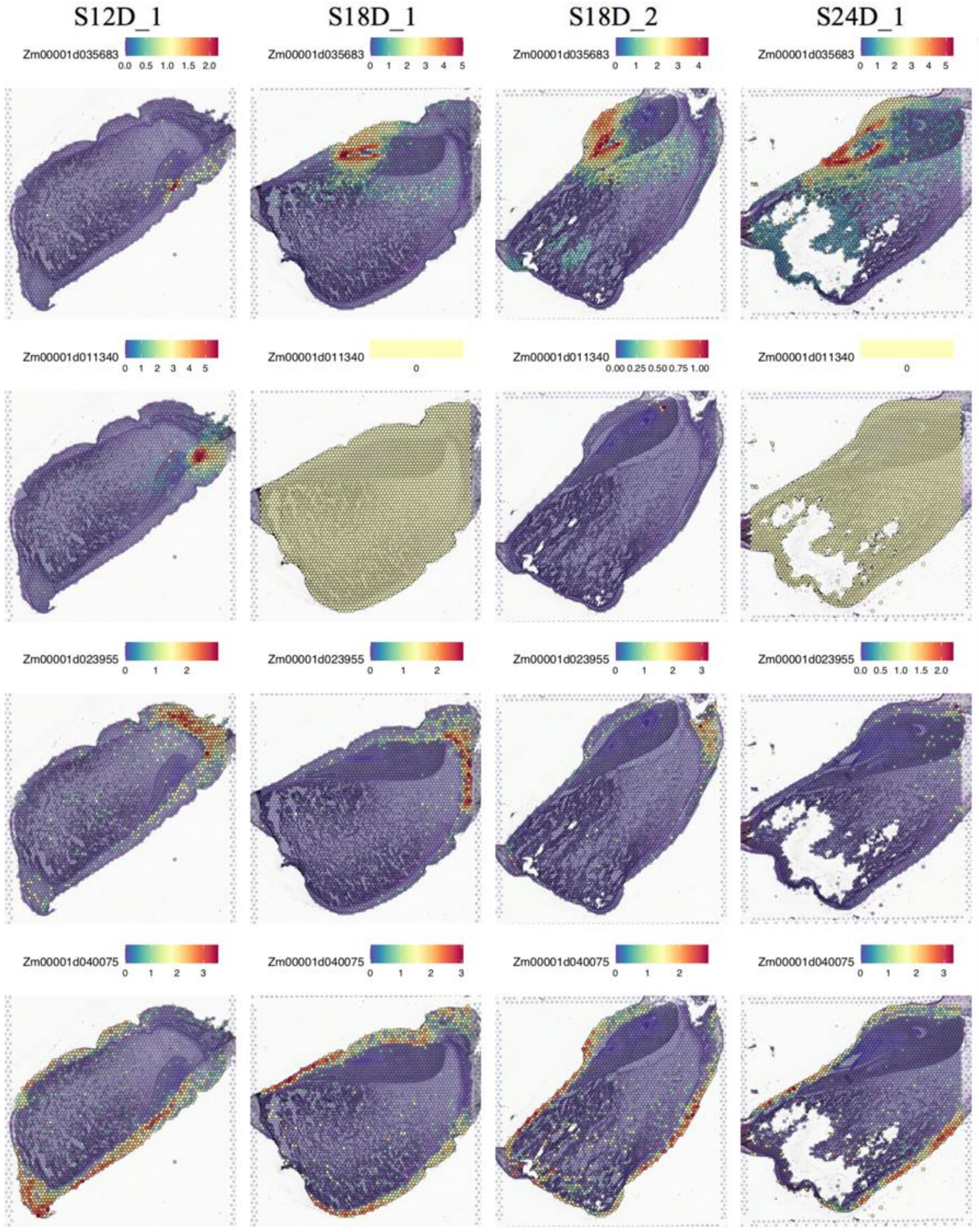
S12D_1
S18D_1
S18D_2
S24D_1
Zm00001d035683
Zm00001d011340
Zm00001d023955
Zm00001d040075

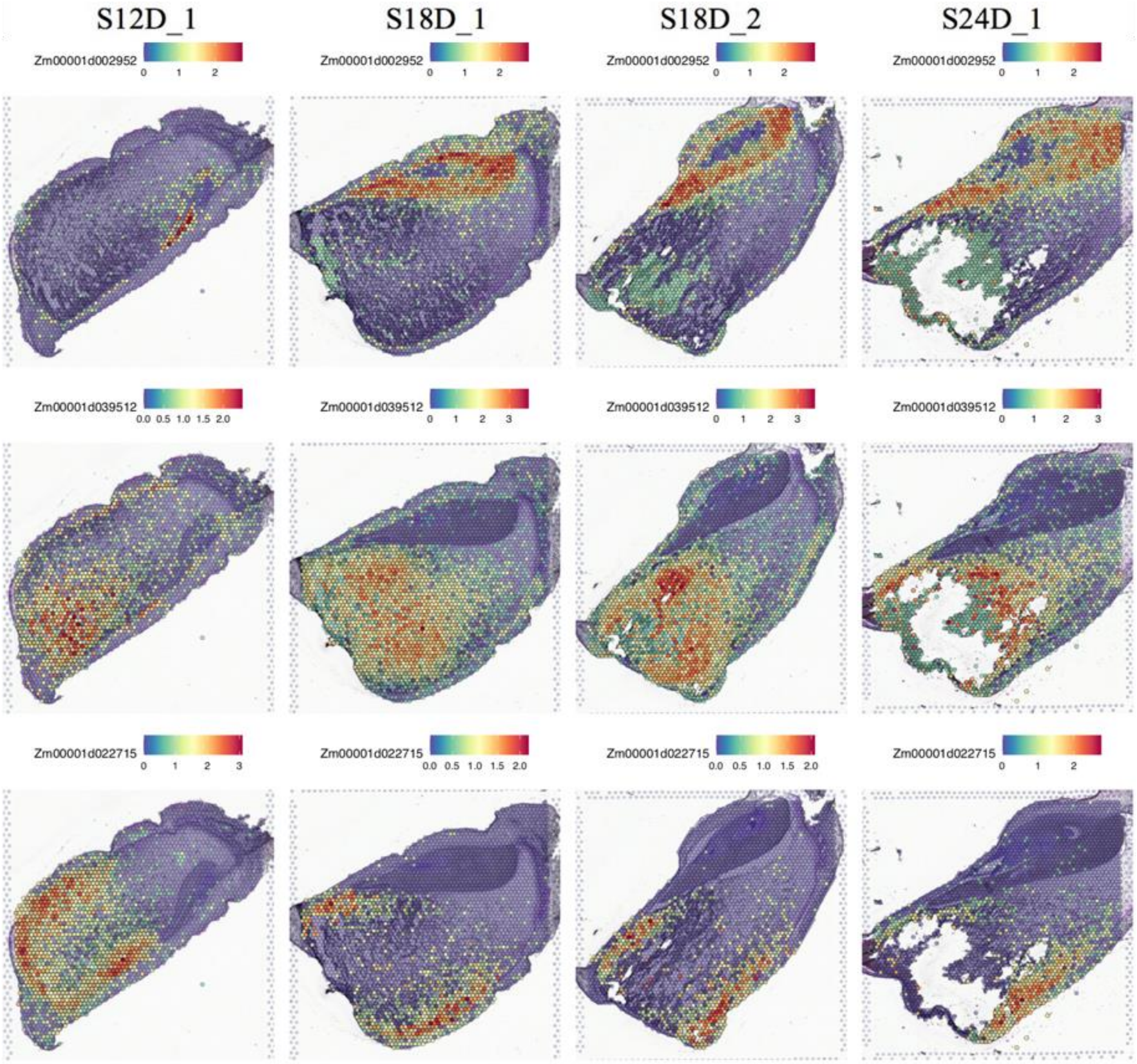

Figure S13. Representatives of electronical RNA in situ hybridization using newly defined markers.

We selected one marker from each compartment as following: non-specific lipid-transfer protein in AL (Zm00001d007299); potassium transporter in BETL (Zm00001d036784); sorbitol dehydrogenase in CZ (Zm00001d031727); phosphate transporter in EAS(Zm00001d018445); subtilisin-chymotrypsin inhibitor in EM (Zm00001d035683); acanthoscurrin in ESR (Zm00001d011340); MAD in PC (Zm00001d023955); glycosyltransferase in PE (Zm00001d040075); aquaporin in SCU (Zm00001d002952); hexokinase in SE (Zm00001d039512); unknown function in VE (Zm00001d022715).

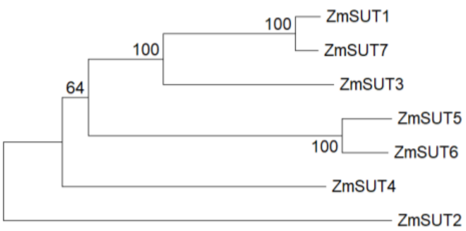

Figure S14. The phylogenetic tree for sucrose transporters (SUTs).

The phylogenetic tree was constructed on MEGA11 using amino acid sequences of SUT1, SUT2, SUT3, SUT4, SUT5, SUT6 and SUT7 with default parameters.

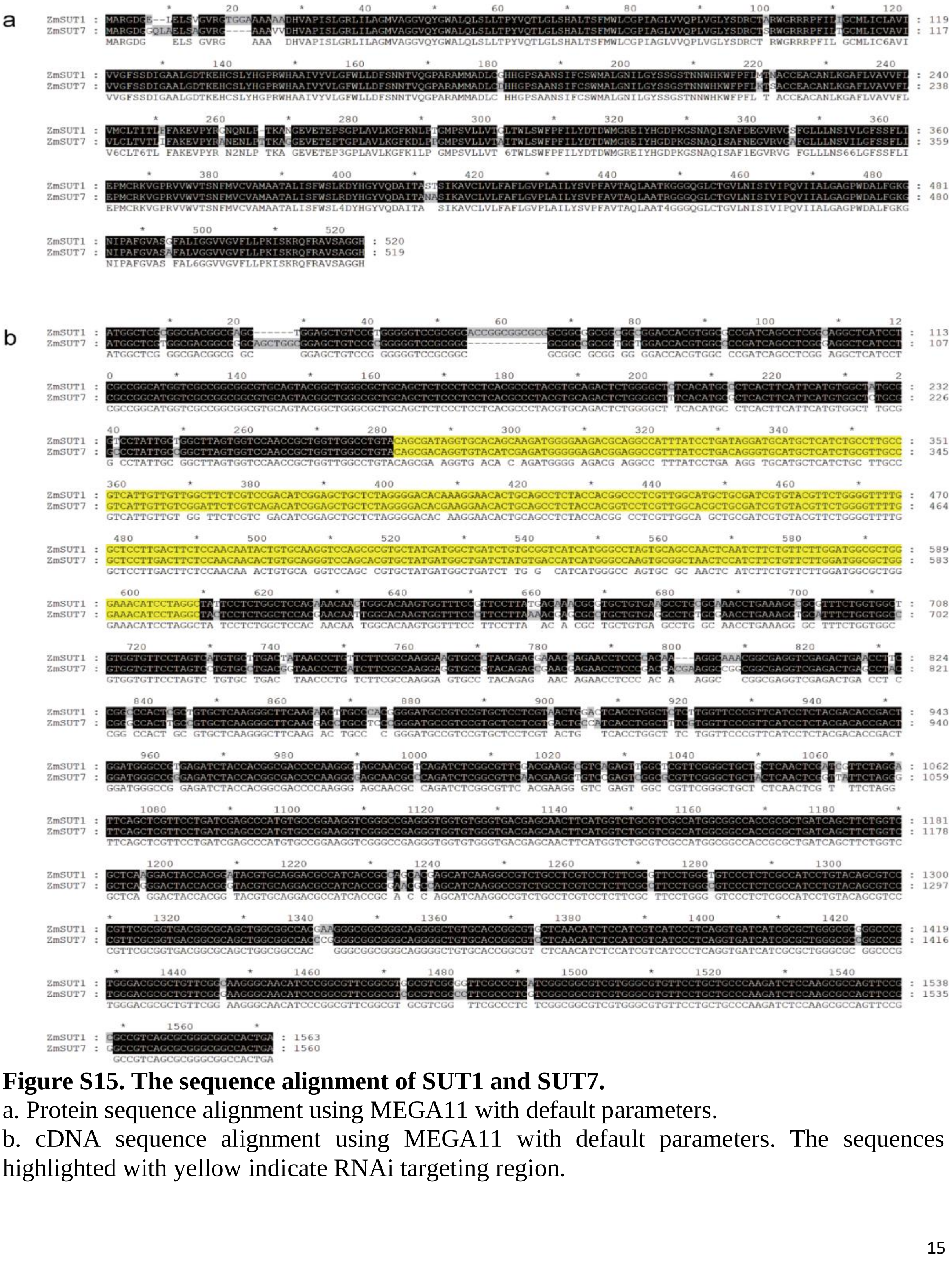

Figure S15. The sequence alignment of SUT1 and SUT7.

a. Protein sequence alignment using MEGA11 with default parameters.

b. cDNA sequence alignment using MEGA11 with default parameters. The sequences highlighted with yellow indicate RNAi targeting region.

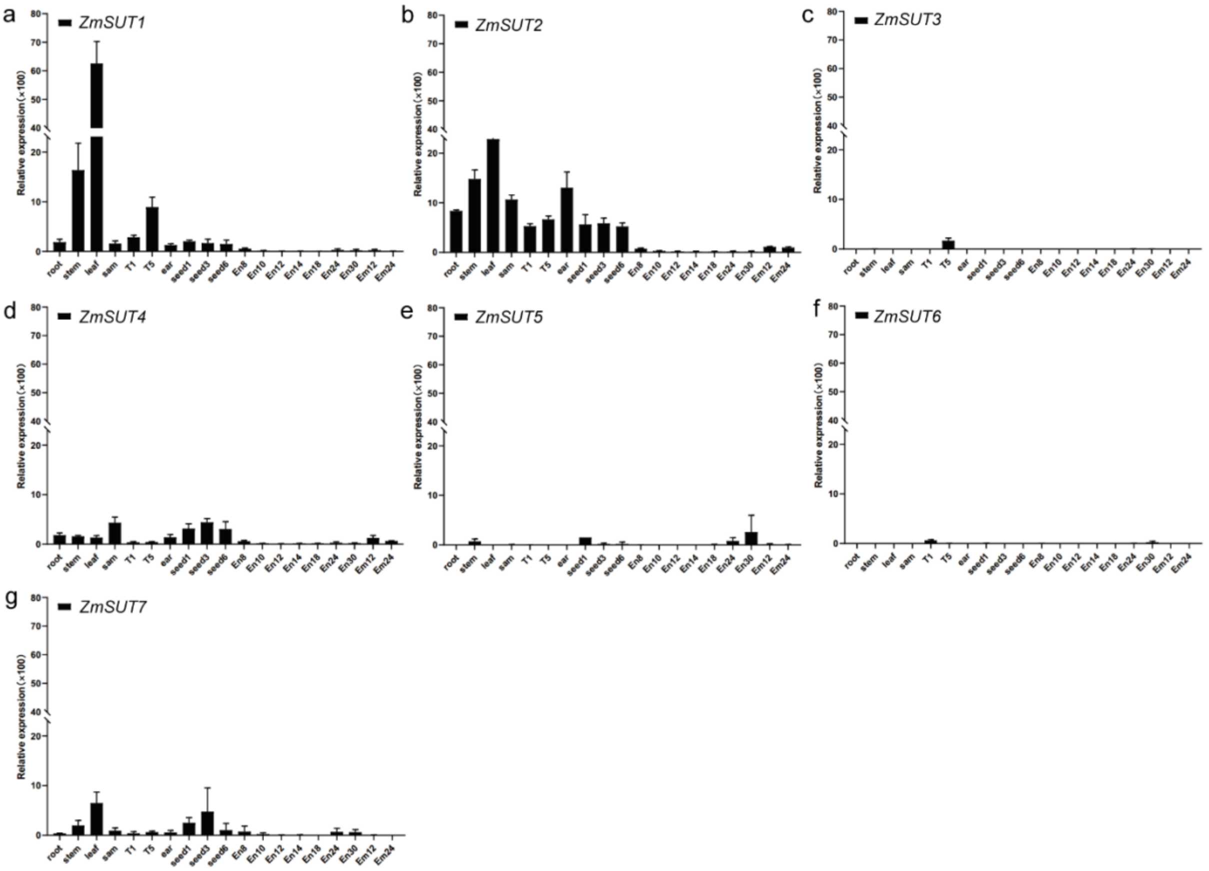

Figure S16. The quantification of SUT gene family using qRT-PCR.

Table S1. The technology comparison of quantifying gene expression using next-generation sequencing in plant science.

Type	Strength	Weakness	Application	Reference
**bulk RNA-seq**	cost-effective	lose spatial content	average gene expression of mixed cell population	Chen, 2014; Yi, 2019
**LCM RNA-seq**	keep spatial information	require prior knowledge and tissue isolation using laser capture microdissection; be a laborious procedure and limited to the yield and purity of the targeted cells; unable to dissect surrounding similar tissues from each other	average gene expression of the same cell population	Zhan, 2015; Doll, 2020; Zhang, 2019
**single-cell RNA-seq**	reach gene expression at single-cell level	require the formation of protoplasts that is challenging for plant cells due to the existence of cell wall; lose spatial information; rely on known molecular markers to reconstruct the cell identity	gene expression of single cell	Zhang, 2019; Zhang, 2021
**spatial transcriptomics**	spatially resolved, high-resolution and high-throughput analyses of average gene expression of few cells captured by a barcoded spot	the gene expression is not equal but close to the sing cell level	high-throughput spatial gene expression profiles and define cell heterogeneity	Giacomello, 2017

Table S2. The statistics of sequencing data.

Raw reads mean the numbers of generated reads from captured tissues. Reads/Spot indicates the number of reads per spot using barcode linkage. nUMI means the number of unique transcripts per spot identified by unique molecular identifiers (UMI). nGene is the number of detected genes per spot. Mit is the sequence percentage from mitochondria. Spots mean the spot number with reads because not all spots have mounted tissue cells in the capture area, leaving ~50% empty out of the total of 5,000 spots.

Sample ID	Raw reads	Reads/Spot	nUMI	nGene	Mit	Spots
**S12D_1**	425,212,087	176,951	11,049	3,655	0.39%	2,403
**S18D_1**	413,460,518	136,052	10,901	3,573	0.47%	3,039
**S18D_2**	453,568,716	159,147	12,257	3,912	0.73%	2,850
**S24D_1**	394,951,614	151,439	11,351	3,792	1.28%	2,608
**Sum**	1,687,192,935	623,589	45,558	14,931	2.88%	10,900

Table S3. The summary of marker genes for 25 clusters from Spatial transcriptomics and the literature including RNA-seq from manual dissection or laser microdissection, as well as experimental in situ hybridization.

Cluster ID	Marker genes from Spatial transcriptomics	Marker genes from RNA-seq using manual or laser laser microdissection	Marker genes using experimental in situ hybridization
C0	Zm00001d023955, Zm00001d052136, Zm00001d027854	Zm00001d006669	Zm00001d027854, Zm00001d052136
C1	Zm00001d008925, Zm00001d027291, Zm00001d017438	Zm00001d008925, Zm00001d027291, Zm00001d017438	-
C2	Zm00001d037382, Zm00001d015515, Zm00001d024522	Zm00001d037382, Zm00001d015515, Zm00001d024522	-
C3	Zm00001d024996, Zm00001d042541, Zm00001d018629	Zm00001d024996, Zm00001d042541, Zm00001d018629	-
C4	Zm00001d046596	Zm00001d046596	-
C5	Zm00001d046126	Zm00001d046126	Zm00001d046126
C6	Zm00001d020395, Zm00001d033447	Zm00001d020395, Zm00001d033447	-
C7	Zm00001d049179, Zm00001d048643	Zm00001d049179, Zm00001d048643	-
C8	Zm00001d033905, Zm00001d041489, Zm00001d035439	Zm00001d033905, Zm00001d041489	Zm00001d035439
C9	Zm00001d041822, Zm00001d052759, Zm00001d019277	Zm00001d041822, Zm00001d052759	Zm00001d041822
C10	Zm00001d007299, Zm00001d012572, Zm00001d020938	Zm00001d012572, Zm00001d020938	Zm00001d046599, Zm00001d012572
C11	Zm00001d050577, Zm00001d017285, Zm00001d037439	Zm00001d050577, Zm00001d017285, Zm00001d037439	Zm00001d050577, Zm00001d017285, Zm00001d037439
C12	Zm00001d009292, Zm00001d013159	Zm00001d009292, Zm00001d013159	Zm00001d009292, Zm00001d013159
C13	Zm00001d003677, Zm00001d013159	Zm00001d003677, Zm00001d013159	Zm00001d013159
C14	Zm00001d029696, ENSRNA049478426	-	Zm00001d037498
C15	Zm00001d051653, Zm00001d033714, Zm00001d043610	-	Zm00001d009646
C17	Zm00001d028714	Zm00001d028714	-
C16	Zm00001d018727	Zm00001d018727	-
C18	Zm00001d013956, Zm00001d022464, Zm00001d040127	-	-
C19	Zm00001d048808	Zm00001d048808	Zm00001d048808
C20	Zm00001d020591	Zm00001d020591	Zm00001d020591
C21	Zm00001d030855, Zm00001d035760	Zm00001d030855, Zm00001d035760	Zm00001d030855, Zm00001d035760
C22	Zm00001d045937	Zm00001d045937	Zm00001d045937
C23	Zm00001d025343	-	-
C24	Zm00001d027819, Zm00001d020780, Zm00001d011342	Zm00001d027819, Zm00001d020780, Zm00001d011342	Zm00001d027819

Table S4. Spot number in clusters after dimensionality reduction.
The number is the spot number within the corresponding cluster.

Cluster	S12D_1	S18D_1	S18D_2	S24D_1	Sum	Compartment	Genotype
**c0**	181	201	101	19	502	PC	maternal
**c1**	196	192	130	150	668	PE	maternal
**c2**	155	88	111	84	438	PE	maternal
**c3**	120	45	67	65	297	PE	maternal
**c4**	50	147	176	210	583	SCU	embryo
**c5**	9	78	64	120	271	SCU	embryo
**c6**	21	92	71	118	302	SCU	embryo
**c7**	20	56	79	110	265	EM	embryo
**c8**	27	29	69	112	237	EM	embryo
**c9**	72	70	72	12	226	BETL	endosperm
**c10**	150	80	93	79	402	AL	endosperm
**c11**	32	51	77	58	218	EAS	endosperm
**c12**	61	98	107	89	355	CZ	endosperm
**c13**	97	91	70	58	316	CZ	endosperm
**c14**	245	178	176	137	736	SE	endosperm
**c15**	108	177	230	155	670	SE	endosperm
**c16**	140	455	291	293	1179	SE	endosperm
**c17**	146	181	115	95	537	SE	endosperm
**c18**	54	82	169	122	427	SE	endosperm
**c19**	119	198	201	140	658	VE	endosperm
**c20**	155	125	113	119	512	VE	endosperm
**c21**	89	161	133	147	530	VE	endosperm
**c22**	80	98	85	49	312	VE	endosperm
**c23**	58	66	50	67	241	VE	endosperm
**c24**	18	0	0	0	18	ESR	endosperm
**Sum**	2403	3039	2850	2608	10900		

Table S5. GO enrichments for WGCNA modules.

Module	Colour	Population	Gene numbers	GO enrichment
**M1**	black	EAS	151	carbohydrate transport
**M2**	blue	SCU	443	fatty acid biosynthetic process
**M3**	brown	BETL	375	ion transmembrane transporter activity
**M4**	green	EM	208	DNA replication
**M5**	greenyellow	PE	115	cell wall biogenesis; oxylipin metabolic process
**M6**	magenta	AL	131	cellular amino acid metabolic process
**M7**	pink	VE	90	nutrient reservoir activity
**M8**	purple	CZ	112	cell-cell junction; plasmodesma; symplast; apoplast
**M9**	red	SE	144	starch biosynthetic process
**M10**	turquoise	PC	220	positive regulation of transcription
**M11**	yellow	ESR	337	coenzyme binding

Table S6. The comparison of defined markers between LCM and Spatial transcriptome. The markers from previous study of laser-capture microdissection (LCM) was utilized from Zhan et al. 2015.

	ST													LCM		
Gene ID	AL	BETL	CZ	EAS	ESR	PC	PE	EM	SCU	SE	VE	max	Population	Highest CS score	Compartment	Gene function
Zm00001d012572	62.28	3.69	3.4	0.18	44.28	10.93	10.12	0.84	0.7	1.01	3.3	62.28	AL	0.51	AL	aleurone9
Zm00001d015569	4.74	0.22	0.6	0.53	0.78	0.94	0.72	0.45	0.51	0.13	0.33	4.74	AL	0.70	AL	vacuolar H+-translocating inorganic pyrophosphatase
Zm00001d000161	0.19	47.23	2.84	0.4	0.33	4.33	0.17	0.08	0.23	0.08	0.07	47.23	BETL	0.97	BETL	basal endosperm transfer layer 4 (BETL4)
Zm00001d003776	0.08	30.57	0.75	0.18	1.56	3.08	0.15	0.02	0.08	0.05	0.04	30.57	BETL	0.95	BETL	sucrose degrading enzyme (CWIN2, mn1)
Zm00001d009292	0.41	1	6.3	2.4	0.17	0.43	0.64	0.07	0.09	3.21	5	6.3	CZ	0.70	CZ	PLATZ transcription factor (floury3)
Zm00001d021289	1.77	2.77	9.47	3.96	0.28	0.83	1.66	0.19	0.19	5.71	9.06	9.47	CZ	0.70	CZ	late emryonic protein
Zm00001d002705	0.2	0.25	0.45	2.56	0.06	0.13	0.11	0.01	0.11	0.04	0.28	2.56	EAS	0.65	CSE (VEandSE)	ATP dependent copper transporter
Zm00001d015914	0.16	0.08	0.22	1.86	0	0.33	0.2	0.25	0.37	0.03	0.04	1.86	EAS	0.62	EM (EMandSCU)	sugars will eventually be exported transporter4b
Zm00001d037985	0.05	0.26	0.74	1.3	0	0.55	4.98	29.36	5.36	0.61	0.34	29.36	EM	0.94	EM (EMandSCU)	late EMryonic protein; EMryo specific protein5
Zm00001d043049	0.03	0.17	0.6	2.12	1.22	0.31	4.5	19.05	2.64	0.34	0.2	19.05	EM	0.81	EM (EMandSCU)	non-specific lipid-transfer protein
Zm00001d027819	0.02	5.94	1.22	0.07	31.44	0.59	0.2	0.04	0.04	0.01	0.01	31.44	ESR	0.99	ESR	emryo surrounding region 2
Zm00001d053112	0.06	7.56	1.19	0.07	13.61	1.47	0.14	0.03	0.06	0.02	0.02	13.61	ESR	0.99	ESR	emryo surrounding region 6
Zm00001d025373	0	1.21	0.06	0.01	0	1.79	0.02	0	0.02	0	0	1.79	PC	0.32	PC	aluminum-activated malate transporter 8
Zm00001d048611	0.78	0.8	0.12	0.02	0.06	7.86	4.56	0.06	0.02	0.38	0.06	7.86	PC	0.85	PC	metallothionein-like protein 1B
Zm00001d020583	0.2	0.03	0.02	0.03	0	0.39	1.01	0.03	0.04	0.02	0.02	1.01	PE	0.95	PE	galactosyltransferase family protein
Zm00001d038476	0.15	0.02	0.02	0.02	0	0.11	1.25	0.01	0.01	0.05	0.03	1.25	PE	0.89	PE	alpha expansin5
Zm00001d026317	0.03	0.35	0.5	1.58	1.67	0.43	0.45	1.62	5.25	0.18	0.14	5.25	SCU	0.94	EM (EM andSCU)	Transcription factor PIF4
Zm00001d019504	1.02	0.26	1.15	3.64	8.56	1.26	2.18	7.47	10.92	0.77	0.42	10.92	SCU	0.91	EM (EM and SCU)	plasma memrane associated protein;tonoplast intrinsic proteins
Zm00001d045042	9.82	0.72	7.85	3.04	0.11	2.21	8.02	1.59	0.64	67.57	36.04	67.57	SE	0.89	CSE (VEandSE)	sucrose synthase 1 (sh1)
Zm00001d050032	1.77	0.2	1.34	0.47	0.44	0.79	2.12	0.29	0.11	19.84	10.4	19.84	SE	0.88	CSE (VEandSE)	glucose-1-phosphate adenylyltransferase (bt2)
Zm00001d020592	454.82	15.17	191.28	153.4	3.22	12.36	198.52	9.51	19.3	505.1	771.71	771.71	VE	0.96	CSE (VEandSE)	27-kD γ-zein
Zm00001d005793	268.27	7.19	80.4	162.01	2.06	7.6	120.84	6.38	16.24	366.71	483	483	VE	0.95	CSE (VEandSE)	16-kD γ-zein (mucronate1)

Table S7. Primers for RNA in situ hybridization.

Figure	Gene ID	Name	Description	Primer-F	Primer-R
Fig.2a	Zm00001d048847	19KD α-zein	zpl2a - zein polypeptidesL2a	19KD-F1: CACAAGCTCCTATAGCTTCCCTT	19KD-R1: AATGTTGCTGTGTCAAGAAGGC
Fig.2c	Zm00001d049476	19KD α-zein	z1A-1 - alpha zein 19kDa A-1	19KD-F2: CTGCTACCGCGACCATTTTC	19KD-R2: GTGTCAAGAAGGCAGCAGGG
Fig.2e	Zm00001d048848	19KD α-zein	z1A-3 - alpha zein 19kDa A-3	19KD-F3: TCCTCCAACAATCATCAGCCCTA	19KD-R3: TTGGTAGAACACTGCTGGGTTTG
Fig.2g	Zm00001d018727	psk1	phytosulfokine peptide precursor 1	ST19-F1: CATTCATTCCTTCTTCCATGGCG	ST19-R1: GGCCGGAGACTCTTCTCTTTATT
Fig.2i	Zm00001d031727	sdh1	sorbitol dehydrogenase homolog1	ZmST10-F2: CGTGGCTGGTTGCCAAGAAC	ZmST10-R2: TCATGTCCTCGCACAGGTTG
Fig.2k	Zm00001d002705	hma5	heavy metal ATPase5	ZmST8-F1: CTTCGTCTCGGAGAACAAAATTACC	ZmST8-R1: AGCTGCTCGAAGAACTGAGTAAA
Fig.2m	Zm00001d002768	ole1	oleosin 1	ZmST11-F2: CTCGTAGTCGTAGCTCAAGCATC	ZmST11-R1: CTCCGATCAAAGAGAGACGCATA
Fig.2o	Zm00001d043049	plt3	putative lipid-transfer protein 3	ZmST12-F2: CCTGCCCTGCCATCATATCG	ZmST12-R2: TGCAGTTAACGTTGGTGCTG
Fig.2q	Zm00001d052136	Acco20	oxidase20/1-aminocyclopropane-1-carboxylate oxidase 20	ZmST5-F1: AAGTGATGAAGCAGTTCGCATC	ZmST5-R1: CATTCCACGATACACGCATAACC
Fig.2s	Zm00001d038558	Cc3	Corn Cystatin (CC)3	ZmST6-F1: GATCGATGGCTGAGGTACACAAT	ZmST6-R1: AGGCTTTCAGATTCCTTAGCACA
Fig.2u	Zm00001d000161	betl4	basal endosperm transfer layer4	ZmST17-F1: TTCACGCGTTCAAAGATGCG	ZmST17-R1: GCCTCCGAACTCCATTAGCA
Fig.2w	Zm00001d007299	LTP	probable non-specific lipid-transfer protein 2	ST23-F1: CGACCAAACTAACAACAGCTCAG	ST23-R1: AACAGAACGTCCTACATGATCCA

Table S8. Primers for qRT-PCR.

Name	Description	Primer-F	Primer-R
actin	actin	GCTACGAGATGCCTGATGGTC	CCCCCACTGAGGACAACG
sut1	sucrose transporter 1	GCGATAGGTGCACAGCAAGA	TCGGACGAGAAGCCAACAAC
sut2	sucrose transporter 2	GCCCTGGGAAACATACTTGGA	TGTCAAGAAGAAAGGCAGACTTA
sut3	sucrose transporter 3	GTGATGGGATTCTGGCTGCT	CACGACACGAAGATGGCGTT
sut4	sucrose transporter 4	TCGCTGAAGAGAGCCCACTA	GGTTCATTTGATGCATGGGCA
sut5	sucrose transporter 5	CGAGTGGGCCACCTTGAACAT	GACCATGCAGGCCAAGAAGAG
sut6	sucrose transporter 6	GCAGACAGGGTTTGGTCTCT	CTTGTGAGGGATCCGAAAAGC
sut7	sucrose transporter 7	CAGCTGGCGGAGCTGTC	GCCCAGCCGTACTGCAC

Exploring the online resource and visualizing electronical RNA in situ hybridization

The website (http://119.78.67.206:3838/) is dedicated to visualizing electronical RNA in situ hybridization images of the tissue sections from the maize inbred line W64A. On the main interface of the webpage, you will find a demonstration examples that utilizes known marker gene.

To retrieve specific electronical in situ images, simply enter a gene ID (e.g., Zm00001d012572) into the search box. After submitting the gene ID, it may take approximately 10 seconds to 2 minutes, depending on your internet connection, to retrieve the results. You will receive four electronical in situ images corresponding to different developmental stages of the maize kernel.

The "slice1" image represents the maize kernel at 12 days after pollination (DAP), while "slice1.1" and "slice1.2" are two biological replicates, representing the maize kernel at 18 DAP. Additionally, "slice1.3" represents the maize kernel at 24 DAP.

Please note that the gene ID used is based on the B73v4 reference genome. If you have gene IDs from other versions, please visit maizeGDB to convert them accordingly.
