# Supplementary material for: Spatial transcriptomics uncover sucrose post-phloem transport during maize kernel development
Source: Nat Commun. 2023 Nov 8;14:7191. doi: 10.1038/s41467-023-43006-7 (PMC10632454; doi:10.1038/s41467-023-43006-7)
Supplement: Supplementary file 3 — Description of Additional Supplementary Files [file 41467_2023_43006_MOESM3_ESM.pdf]

Description of Additional Supplementary File

Supplementary Data 1. The expressed genes in at least one compartment.
There are 26,161 genes expressed in at least one of the 11 compartments after pooling all four sections. The number indicates UMI index, which is normalized by the total expressed transcripts distinguished by UMI in a spot and multiplied by a scale factor of 10,000.

Supplementary Data 2. The gene list existed in WGCNA modules.
Weighted correlation network analysis (WGCNA) is used for finding clusters (modules) of highly correlated genes

Supplementary Data 3. The details of differentially expressed genes.
Differential gene analysis is conducted over the 11 compartments. After redundancy removal, there are 2,992 genes defined as differentially expressed genes.

Supplementary Data 4. The details for the defined marker genes.
The marker gene is defined as the significant gene-gene connectivity in WGNCA analysis, belonging to the members of differentially expressed genes and a minimal expression level of 1 (UMI index).
