# Supplementary material for: Spatial transcriptomics uncover sucrose post-phloem transport during maize kernel development
Source: Nat Commun. 2023 Nov 8;14:7191. doi: 10.1038/s41467-023-43006-7 (PMC10632454; doi:10.1038/s41467-023-43006-7)
Supplement: Supplementary file 8 — Reporting Summary [file 41467_2023_43006_MOESM8_ESM.pdf]

nature portfolio

Corresponding author(s): Wenqin Wang, Yongrui Wu
Last updated by author(s): Oct 12, 2023

Reporting Summary

Nature Portfolio wishes to improve the reproducibility of the work that we publish. This form provides structure for consistency and transparency in reporting. For further information on Nature Portfolio policies, see our Editorial Policies and the Editorial Policy Checklist.

Statistics

For all statistical analyses, confirm that the following items are present in the figure legend, table legend, main text, or Methods section.

n/a	Confirmed	
☐	☒	The exact sample size (*n*) for each experimental group/condition, given as a discrete number and unit of measurement
☐	☒	A statement on whether measurements were taken from distinct samples or whether the same sample was measured repeatedly
☐	☒	The statistical test(s) used AND whether they are one- or two-sided<br>*Only common tests should be described solely by name; describe more complex techniques in the Methods section.*
☒	☐	A description of all covariates tested
☐	☒	A description of any assumptions or corrections, such as tests of normality and adjustment for multiple comparisons
☐	☒	A full description of the statistical parameters including central tendency (e.g. means) or other basic estimates (e.g. regression coefficient) AND variation (e.g. standard deviation) or associated estimates of uncertainty (e.g. confidence intervals)
☐	☒	For null hypothesis testing, the test statistic (e.g. *F*, *t*, *r*) with confidence intervals, effect sizes, degrees of freedom and *P* value noted<br>*Give P values as exact values whenever suitable.*
☒	☐	For Bayesian analysis, information on the choice of priors and Markov chain Monte Carlo settings
☒	☐	For hierarchical and complex designs, identification of the appropriate level for tests and full reporting of outcomes
☒	☐	Estimates of effect sizes (e.g. Cohen's *d*, Pearson's *r*), indicating how they were calculated

Our web collection on statistics for biologists contains articles on many of the points above.

Software and code

Policy information about availability of computer code

Data collection	Semi-thin section imaging: ZEISS Axio Zoom V16 385 microscope;<br>In-situ hybridization sections were observed and imaged using: DM2500 Microscope (Leica, Germany);<br>HE staining slices imaging was performed:3D HISTECH 397 Pannoramic MIDI FL at 40×resolution;<br>kernels phenotype imaging: DM2500 Microscope (Leica, Germany);<br>Spatial Transcriptome Sequencing: Illumina novaseq6000 (Illumina).
Data analysis	Softwares:<br>Space Ranger V1.2.0 (spatial transcriptomic sequencing mapping), Adobe Illustrator CS6 (compiling final figures), GraphPad Prism v8.0.2 (graph chart), SnapGene (sequence analysis)<br><br>R(v4.1.1) and R packages:<br>The public Seurat R package was used to analyze the transcriptomic data. Such as: Seurat V4.0.3, clusterProfiler V3.18.1, dplyr V1.0.7.<br>Reference: Hao*, Hao*, et al., Cell 2021 [Seurat v4].<br><br>The code were deposited in a Github repository:<br>https://github.com/wwq413/SpatialTranscriptomics/

For manuscripts utilizing custom algorithms or software that are central to the research but not yet described in published literature, software must be made available to editors and reviewers. We strongly encourage code deposition in a community repository (e.g. GitHub). See the Nature Portfolio guidelines for submitting code & software for further information.

Data

Policy information about availability of data

All manuscripts must include a data availability statement. This statement should provide the following information, where applicable:

- Accession codes, unique identifiers, or web links for publicly available datasets
- A description of any restrictions on data availability
- For clinical datasets or third party data, please ensure that the statement adheres to our policy

The raw sequencing data of spatial Transcriptomics generated in this study have been deposited in the NCBI Sequence Read Archive under the Bioproject of PRJNA1031340 with the accession code of SRX22238644-SRX22238647. Source data supporting the results of this study are provided. Any other data or materials that support the finding of this study are available for the corresponding authors upon request.

Field-specific reporting

Please select the one below that is the best fit for your research. If you are not sure, read the appropriate sections before making your selection.

[x] Life sciences [ ] Behavioural & social sciences [ ] Ecological, evolutionary & environmental sciences

For a reference copy of the document with all sections, see nature.com/documents/nr-reporting-summary-flat.pdf

Life sciences study design

All studies must disclose on these points even when the disclosure is negative.

Sample size	No statistical methods were used to predetermine sample size. Sample sizes were estimated based on preliminary experiments and previously published results. Kernels selected from the middle region of ears with uniform kernel set were used for photography and kernel trait measurement, and at least 8 kernels were measured for each ear. For in situ hybridization, more than 50 developing kernels were randomly dissected and hybridized.
Data exclusions	No data was excluded from the analyses.
Replication	All experiments in this study were repeated independently at least three times.
Randomization	All samples were arranged randomly into experimental groups.
Blinding	For molecular biology experiments, bias could not be introduced since samples were treated identically and collected randomly. No analyses required being blind to groups.

Reporting for specific materials, systems and methods

We require information from authors about some types of materials, experimental systems and methods used in many studies. Here, indicate whether each material, system or method listed is relevant to your study. If you are not sure if a list item applies to your research, read the appropriate section before selecting a response.

Materials & experimental systems

n/a	Involved in the study	
[x]	[ ]	Antibodies
[x]	[ ]	Eukaryotic cell lines
[x]	[ ]	Palaeontology and archaeology
[x]	[ ]	Animals and other organisms
[x]	[ ]	Human research participants
[x]	[ ]	Clinical data
[x]	[ ]	Dual use research of concern

Methods

n/a	Involved in the study	
[x]	[ ]	ChIP-seq
[x]	[ ]	Flow cytometry
[x]	[ ]	MRI-based neuroimaging
